# Reactive oxygen species enhance rAAV transduction by promoting its escape from late endosomes

**DOI:** 10.1186/s12985-023-01964-w

**Published:** 2023-01-07

**Authors:** Xiaoping Huang, Xiao Wang, Yanxuan Ren, Pingzhang Gao, Wentao Xu, Xiaolan Xie, Yong Diao

**Affiliations:** 1 College of Chemical Engineering and Materials Sciences, Quanzhou Normal University, Quanzhou, China; 2 School of Medicine, Huaqiao University, Quanzhou, China

**Keywords:** Endosome, ROS, rAAV, Cathepsin B, Cathepsin L

## Abstract

**Background:**

Recent seminal studies have revealed that endosomal reactive oxygen species (ROS) promote rather than inhibit viral infection. Some ROS generators, including shikonin and H_2_O_2_, have the potential to enhance recombinant adeno-associated virus (rAAV) transduction. However, the impact of ROS on rAAV intracellular trafficking remains unclear.

**Methods:**

To understand the effects of ROS on the transduction of rAAV vectors, especially the rAAV subcellular distribution profiles, this study systematically explored the effect of ROS on each step of rAAV intracellular trafficking pathway using fluorescently-labeled rAAV and qPCR quantification determination.

**Results:**

The results showed promoted in-vivo and in-vitro rAAV transduction by ROS exposure, regardless of vector serotype or cell type. ROS treatment directed rAAV intracellular trafficking towards a more productive pathway by upregulating the expression of cathepsins B and L, accelerating the rAAV transit in late endosomes, and increasing the rAAV nucleus entry.

**Conclusions:**

These data support that ROS generative drugs, such as shikonin, have the potential to promote rAAV vector transduction by promoting rAAV’s escape from late endosomes, and enhancing its productive trafficking to the nucleus.

## Introduction

Recombinant adeno-associated virus (rAAV) vectors have revolutionized gene therapy due to their wide tissue tropism, non-pathogenicity, and long-term transgene expression [1, 2]. They have shown great clinical success in several diseases such as Leber’s hereditary optic neuropathy, spinal muscular atrophy type 1, lipoprotein lipase deficiency, and hemophilia A and B [3]. Thus far, at least five rAAV gene medicines have been approved in the European Union and the United States. Despite these remarkable achievements, there are still many gaps in our understanding of these vectors [4, 5]. One of main challenges is that a high dose of AAV vectors is required due to the low transduction efficiency. However, higher vector doses inevitably increase the risks of AAV related side effects, such as immune response that potentially causes liver toxicities after systemic administration. In order to increase their transduction efficiency, self-complementary (sc) rAAV was designed for faster and higher gene expression utilizing a mutated ITR [6], and some pharmacological agents have been screened to enhance rAAV transduction [4, 7, 8]. Nevertheless, a deeper understanding of rAAV cellular fate will play a vital role in improving its transduction efficiency.

It is well known that rAAV must enter the cell and travel to the nucleus for transgene expression. As a result, a deeper understanding of the cellular barriers for successful transduction by rAAV is necessary for optimizing rAAV clinic applications. The rAAV transduction process is extremely complex. Take rAAV2, for example, firstly; it is taken in cells using receptor-dependent endocytosis [9] and traffics via endosomal pathways [10]. Intact rAAV in endosomes undergoes a series of pH-dependent structural transformations essential for endosomal escape and traffic through the cytosol via the cytoskeletal network. Following the endosomal escape, rAAV enters the nucleus through the nuclear pore complex, then undergoes capsid uncoating to release the genome and express the transgene [7]. There are some inefficient processes of rAAV transduction pathway recognized till now, such as intracellular trafficking, second-strand synthesis [8, 11], and nuclear entry[12]. In addition, the development of methods to enhance rAAV transduction has been a continual effort, such as the engineering of novel AAV capsids, rational rAAV genome design, and screening of small molecules that enhance rAAV rAAV transduction [7, 8, 13]

Reactive oxygen species (ROS) are usually considered to be undesirable toxic molecules that are generated under conditions of cellular stress situations. However, recent studies showed that NOX2 oxidase-derived ROS could not eliminate viruses in a way similar to that of bacteria and fungi [14–18]. By contrast, virus-driven endosomal ROS promotes rather than inhibits viral infection [14]. It has been reported that H_2_O_2_ modulates the phosphorylation state of ssD-BP, which is a key player in rAAV genome double-strand synthesis and transduction of rAAV genome [5]. Meanwhile, the efficiency of rAAV nuclear entrance was significantly elevated after H_2_O_2_ treatment [5], suggesting that its intracellular trafficking is also enhanced. It was reported that shikonin, a potential ROS generator, has significantly increased the in vitro and ex vivo transgene expression mediated by rAAV6 vectors in hematopoietic cells, prompting us to investigate how ROS influence the cellular fate of rAAV [1].

Herein, this study describes the results of a systemic study designed to evaluate the impact of ROS (shikonin or H_2_O_2_ as ROS generator) on the transduction efficiency of both single-stranded (ss) and sc rAAV vectors in various cell lines and mice. In addition, the ROS dependence of rAAV transduction was analyzed. Furthermore, almost every step of rAAV transduction was studied, including cell binding, endocytosis, intracellular trafficking from early endosome to the late endosome, escape from the endosome, and nuclear entry, to investigate the effects of ROS on the cellular fate of rAAV vectors. Two endosomal proteases, cathepsins B and L, were discovered to be key players in ROS-enhanced rAAV transduction, especially in the step of rAAV escape from late endosomes.

## Materials and methods

### Cell culture and chemicals

Human embryonic kidney cell line HEK 293-T (Chinese Academy of Sciences Cell Bank, GNHu17) and human cervical cancer cell line Hela (ATCC, CRM-CCL-2, Manassas, VA, USA) were used in this study. We also used human primary hepatocytes LO-2 (Procell Life Science & Technology, HL-7702) in this study. All cells were maintained in monolayer cultures in DMEM or RPMI-1640 supplemented with 1% penicillin–streptomycin (PS, Procell Life Science&Technology Co.,Ltd., Wuhan, China) and 10% fetal bovine serum (FBS). Cells were subject to incubation in a humidified chamber at 37 °C with 5% CO_2_. Bafilomycin A1 (Shanghai YuanYe Bio-Technology, Shanghai, China) solution was prepared in dimethylsulfoxide (DMSO) at the concentration of 10 mM. D-Luciferin (Beyotime, Shanghai, China) was prepared in PBS at 150 mg/mL concentration. In addition, cathepsin B inhibitor CA-074 (Sigma-Aldrich, C5732) and cathepsin L Inhibitor I (Sigma-Aldrich, 219421) were prepared at a concentration of 5 mM in DMSO.

### Recombinant AAV vector generation

CMV promoter was employed in all recombinant AAV vectors as previously described [15]. In brief, three plasmids were transfected into 293 T cells using polyethyleneimine, Linear, molecular weight 25,000 (PEI 25 K, Polysciences). Following 72 h of transfection, cells were collected, freeze-thawed for 3 cycles, and treated with Benzonase (50 U/mL crude lysates) at 37 °C for 60 min. After that, cesium chloride gradient centrifugation was performed to purify viral vectors. Viral vectors were concentrated by ultracentrifugation followed by three PBS washes and finally resuspended in PBS. qPCR was later performed to measure the titers of rAAV vector stocks. Sequences of real time PCR primers were shown below; forward (F), 5′-tgaccctgaagttcatctgc-3′; reverse (R), 5′-gaagtcgtgc tg cttcatgt-3′.

### Plasmids construction

To generate the pcDNA3.1-CTSB and pcDNA3.1-CTSL plasmids, pAAV plasmid was linearized using *Eco*RI and *Hind*III enzymes. CTSB (NM_001908.5) and CTSL (NM_001382757) coding sequences were synthesized. The following primers were utilized for the amplification of CTSB coding sequence: forward, 5′-ttgaattcatgtggcagctctgggcctccctctg-3′; and reverse, 5′-ggaagcttttaga tcttttcccagtactgatcggt-3′. The following primers were utilized for the amplification of CSTL coding sequence; forward, 5′-ccgaattcatgaat cctacactcatccttgctgc-3′; and reverse, 5′-ggaagctttta cgtagccacccat gccccattc tt-3′. PCR amplified CTSB, and CTSL DNA fragments were cloned into pcDNA3.1. pcDNA 3.1 vector containing CTSB and CTSL coding sequences was transformed into Escherichia coli TOP10 strains.

### Recombinant AAV2 vector transduction assay

HeLa, 293 T, and LO-2 cells (1 × 10^4^/well) were seeded in a 96-well plate and incubated for 12 h at 37 °C. Following pretreatment with shikonin or H_2_O_2_ for 4 h, the culture medium was replaced with DMEM containing FBS. Cells were transduced with different multiplicities of infection (MOI) with different rAAV2 vectors for 72 h at 37 °C. GFP expression levels were monitored with IncuCyte cell imaging system (Essen BioScience, MI, USA) and were analyzed by extracting the average fluorescence intensity at three different fields of each well.

### siRNA transfection

Target sequences for siRNAs of CTSB and CTSL were designed and synthesized by GenePharma (Shanghai, China) (Table 1). HeLa cells were transfected with siRNAs specific for CTSB and CTSL. Scrambled siRNA was used as the control. Total cellular or tissue proteins were collected after 24 h of transfection, and gene silencing efficiency was measured by Western-blot (WB) assay. In some assays, Hela cells, were transfected with siRNA for 24 h, were further transduced with rAAV2. The endosomes were isolated, and qPCR analysis was carried out to identify the genome of rAAV2.

**Table 1:** Sequences of siRNA adopted for ablating CTSL and CTSB protein expression

Target siRNA sense and antisense strand sequence (5'–3')	
CTSL siRNA	CCCUCGAAGGACAGAUGUUTTAACAUCUGUCCUUCGAGGGTT
CTSB siRNA	GCCCGACCAUCAAAGAGAUTTAUCUCUUUGAUGGUCGGGCTT
Scrambled siRNA	UUCUCCGAACGUGUCACGUTTACGUGACACGUUCGGAGAATT

### Capsid labeling and confocal microscopy

Confocal imaging of tetramethylrhodamine (TAMRA)-labeled rAAV2 was performed as previously described [16]. Pure rAAV2 virions were incubated with mono-NHS-TAMRA (100 molecules/genome) (Sangon Biotech, Shanghai, China) at room temperature for 45 min. Then SpinOUT™ GT-600 column (G-Biosciences, MO, USA) was used to remove the unconjugated dye. qPCR was performed to determine the titer of the labeled vectors. Regarding confocal imaging experiments, Hela cells were treated with H_2_O_2_ for 4 h, and then TAMRA-labeled rAAV2 was later used to transduce HeLa cells at 2000 vg/cell. The rAAV2 distribution was analyzed at 1 h, 5 h, and 10 h after incubation. Cells were harvested, washed thrice with PBS, and subsequently fixed for 15 min with 4% paraformaldehyde (PFA) at ambient temperature. Cells were rinsed thrice with PBS, followed by a double-distilled H_2_O wash and were labeled using 4,6-diamidino-2-phenylindole (DAPI) (Sigma Aldrich, MO, USA). To analyze the location of rAAV2 virions, we employed the Zeiss LSM 710 spectral confocal laser-scanning microscope (Zeiss, Oberkochen, Germany).

### Fractionation of cytoplasmic and nuclear protein extracts

Cytoplasmic and nuclear fractions were separated from HeLa cells following the previously described protocol [17]. Briefly, 0.01% trypsin was added to rAAV2-*GFP* infected cells, followed by rinsing with PBS five times to remove unabsorbed virus particles. Later, 200 µL hypotonic buffer (10 mmol/L HEPES, pH 7.9 10 mmol/L KCl, 1.5 mmol/L MgCl_2_, 0.5 mmol/L phenylmethanesulfonylfluoride fluoride, 0.5 mmol/L dithiothreitol) was supplemented to each tube and the cells were resuspended and incubated on ice for 5 min. After that, 10% NP-40 (10 µL) was added to each tube, and incubated for 3 min. The cells were observed under a light microscope (Nikon, Tokyo, Japan). After gentle mixing, samples were centrifuged for 5 min at 500 rpm at 4 °C, and the supernatants (cytoplasmic fraction) were collected and preserved on ice. The pellets (nuclear fraction) were rinsed twice with hypotonic buffer (1 mL) twice and preserved on ice. Cytoplasmic fraction and nuclear fraction) were analyzed to determine the purity of each fraction as described previously [18, 19]. The purity of nuclear and cytoplasmic fractions was > 95%.

### In-vivo transduction assays

Animal experiments were carried out following the guidelines from the University of Huaqiao at the School of Medicine Animal Care and Use Committee. To conduct in vivo transduction assays, the shikonin solution was injected intraperitoneally into each mouse (n = 6) at 2 mg/kg body weight, and DMSO was injected intraperitoneally into six mice as the control. Next, ss-rAAV-Fluc (1 × 10^11^ vg/mouse) vectors carrying the fluc gene were injected intravenously into mice, and luciferase expressions were detected on day1, day 3, day 5, day 7, and day 9. Live imaging of rAAV8 vector luciferase activity was depicted in a previous study [16]. In brief, D-luciferin (Beyotime, Shanghai, China) was intraperitoneally injected (150 mg/kg) into each mouse, and an IVIS-Lumina imaging system (FluoView 100, Guangzhou Biolight Biotechnology Co., Ltd) was utilized to determine bioluminescence activity. Living Image software was adopted for quantifying the signal intensity of bioluminescence, which was shown in units of photons/s/cm^2^/steradian.

### rAAV2 binding and internalization analysis

For assessing rAAV2 binding to the cell, cells were inoculated with rAAV2 for 30 min at 4 °C. After incubation (5000 vg/cell rAAV), cells were rinsed thrice with pre-chilled PBS. Afterwards, the TIANamp Virus DNA/RNA Kit (Tiangen, Beijing, China) was employed for extracting total cellular DNA as described in the manufacturer's guidance. qPCR was conducted with the AceQ qPCR SYBR Green Master Mix kit (SYBR Green I, Vazyme Biotechnology, Nanjing, China) using transgene-specific primers to measure the infection efficiency of rAAV as described previously [20]. For internalization assays, cells were infected for 1 h at 4 °C and cultured for 1 h at 37 °C with 5% CO_2_, followed by trypsin treatment to remove those non-internalized surface-bound virions. After washing thrice with pre-chilled PBS, total cellular DNA was extracted, and qPCR was conducted in order to measure total viral DNA using transgene-specific primers.

### Early and late endosome separation

Early and Late endosomes were isolated according to precious description [21, 22]. At first, the cell dish was placed on ice and washed thrice with pre-chilled PBS. It was scraped and transferred into the 15 ml tubes to centrifuge at 200 g–400 g at 4 °C for 5 min. The PBS was removed and 5 mL of Homogenization Buffer being added (HB, Sucrose, 250 mM, EDTA, 1 mM; Imidazole,3 mM; Cycloheximide, 0.03 mM; Phosphatase inhibitors cocktail; Protease inhibitors cocktail) to loosen the cell pellet. Later, the suspension was transferred into the syringe for homogenization. The homogenization was monitored through phase-contrast microscopy. The homogenate was then diluted in HB, followed by 10-min centrifugation at 1600–2000 g at 4 °C. Supernatants were collected, and soluble fractions were transfected into the 13.5-ml ultracentrifuge tube on ice. Later, pre-chilled 18% percoll/sucrose buffer (9 ml) was delivered to generate one layer under the input. Ultracentrifuge tubes were placed into a rotor followed by an additional 1-h ultracentrifugation at 30,000 × *g* at 4 °C. Subsequently, gradient fractions were harvested from the bottom of the ultracentrifuge tube. WB assay was performed on samples obtained in percoll fractions.

### Western-blot (WB) assay

We carried out WB assay according to the previous description [23]. To analyze cell protein, ~ 5 µg WCL sample was isolated using 10% SDS-PAGE, followed by transfer onto PVDF membranes (Millipore, MA, USA). After 12-h blocking using 5% BSA within TBST (150 m NaCl, 20 mmol/L Tris–HCl, pH 7.5) at 4 °C, followed by overnight incubation using specific primary antibodies at 4 °C, which included rabbit anti-CTSB (A19005, ABclonal); rabbit anti-CTSL (A4986, ABclonal); rabbit-EEA1 (#3288, CST), rabbit-RAB7 (#9367, CST); rabbit anti-GAPDH (#5174, CST). Membranes were then rinsed, followed by 1-h incubation using HRP-conjugated anti-rabbit (1:1000; R & D Systems) at an ambient temperature. Later, ECL–plus chemiluminescence substrate (Amersham Pharmacia Biotech, NJ, USA) was employed for detecting protein bands. Image J was utilized to quantify signal intensity.

### Statistical analysis

Results were represented by mean ± SD. Student’s t-test or one-way ANOVA was conducted for data analysis, with *p* < 0.05 indicating the statistical significance. Graphpad v5.0 (GraphPad, San Diego, CA, USA) was adopted for statistical computations.

## Results

### Shikonin increases both ss-rAAV and sc-rAAV transduction in vitro

Recently, several compounds extracted from Traditional Chinese medicine (TCM) have been reported to promote rAAV transduction both in vivo and in vitro, including shikonin, pristimerin, celastrol, and arsenic [1, 16, 24]. Shikonin was found to improve sc-rAAV6 transduction of hematopoietic cells through enhanced ROS formation [1]. This study focused on exploring whether the enhancement is maintained both for ss-rAAV and sc-rAAV, as well as in different types of cells.

**Fig. 1:**
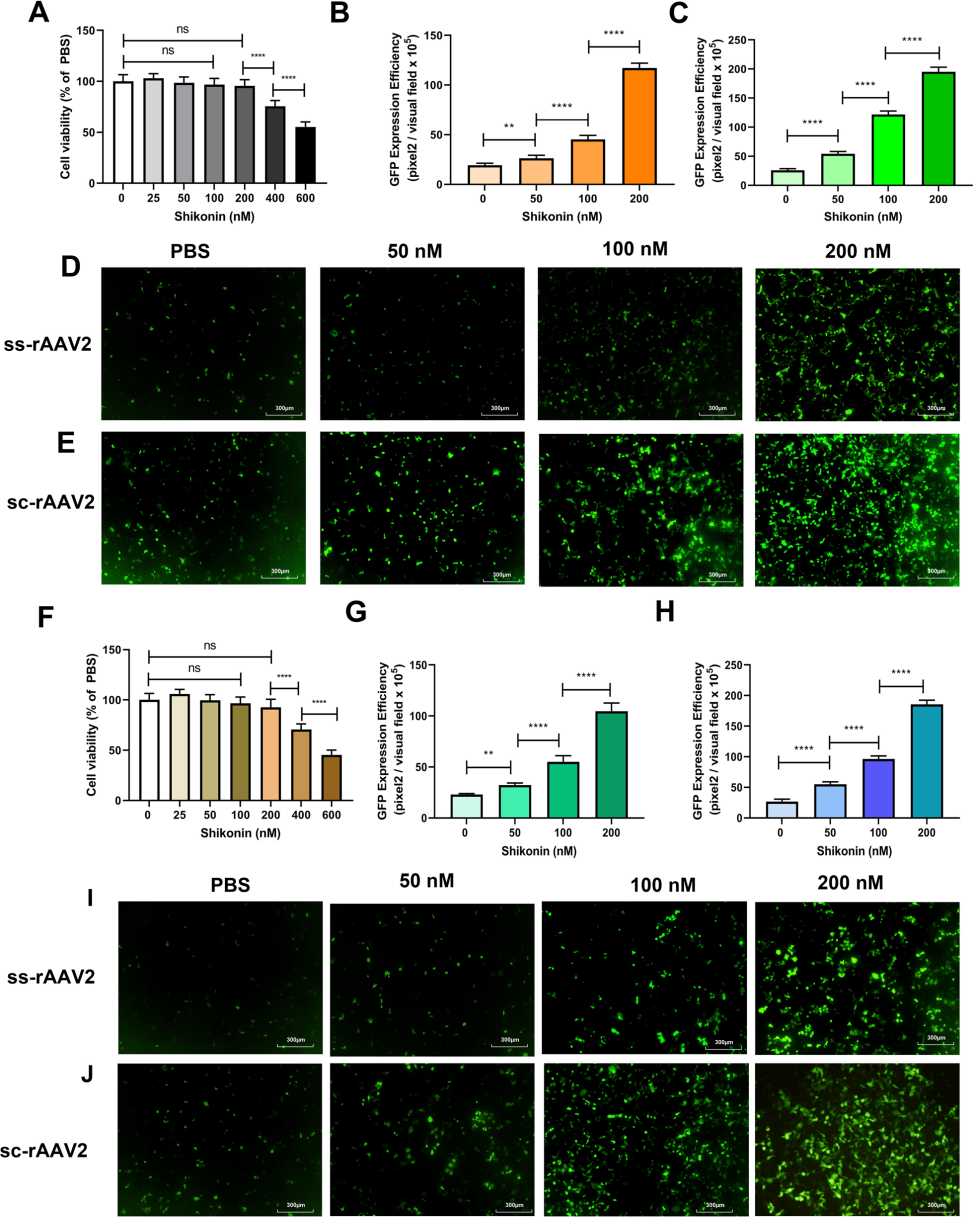
Shikonin pretreatment increased ssAAV2-GFP or scAAV2-GFP transduction in 293 T or HeLa cells. **A** Cell viability results of 293 T exposed to 24-h shikonin treatment at different doses. Quantitation of transduction efficiency of ss-rAAV2 at 500 vg/cell (**B**) or sc-rAAV2 at 300 vg/cell (**C**) in 293 T cells treated with different doses of shikonin. The representative images of ss-rAAV2 (**D**) and sc-rAAV2- mediated **E** transgene expression in pretreated 293 T were measured using fluorescence microscopy after the infection for 48 h. **F** Cell viability results of Hela exposed to 24-h shikonin treatment at various doses. Quantitation of transduction efficiency of ss-rAAV2 at 500 vg/cell (**G**) or sc-rAAV2 at 300 vg/cell (**H**) in Hela cells were treated with different doses of shikonin. The representative images of ss-rAAV2 (**I**) and sc-rAAV2- mediated (**J**) transgene expression in pretreated Hela were measured using fluorescence microscopy after the infection for 48 h. Data are represented by means ± SD (n = 3). ^ns^ *p* > 0.05; **p < 0.01; ****p < 0.0001

The cells were pretreated with shikonin for 4 h, which had little effect on cell viability within the safe concentration range (Fig. 1A, F), and subsequently transduced with either ss-rAAV2 or sc-rAAV2 for 48 h. In 293 T and Hela, 200 nM shikonin pretreatment increased the ss-rAAV2-GFP transduction 6.1 and 4.5 times, compared with PBS (Fig. 1B, D, G, I). Similar to the previous report [1], shikonin pretreatment increased sc-rAAV2-GFP transduction in 293 T and Hela cells (Fig. 1C, E, H, J).

**Fig. 2:**
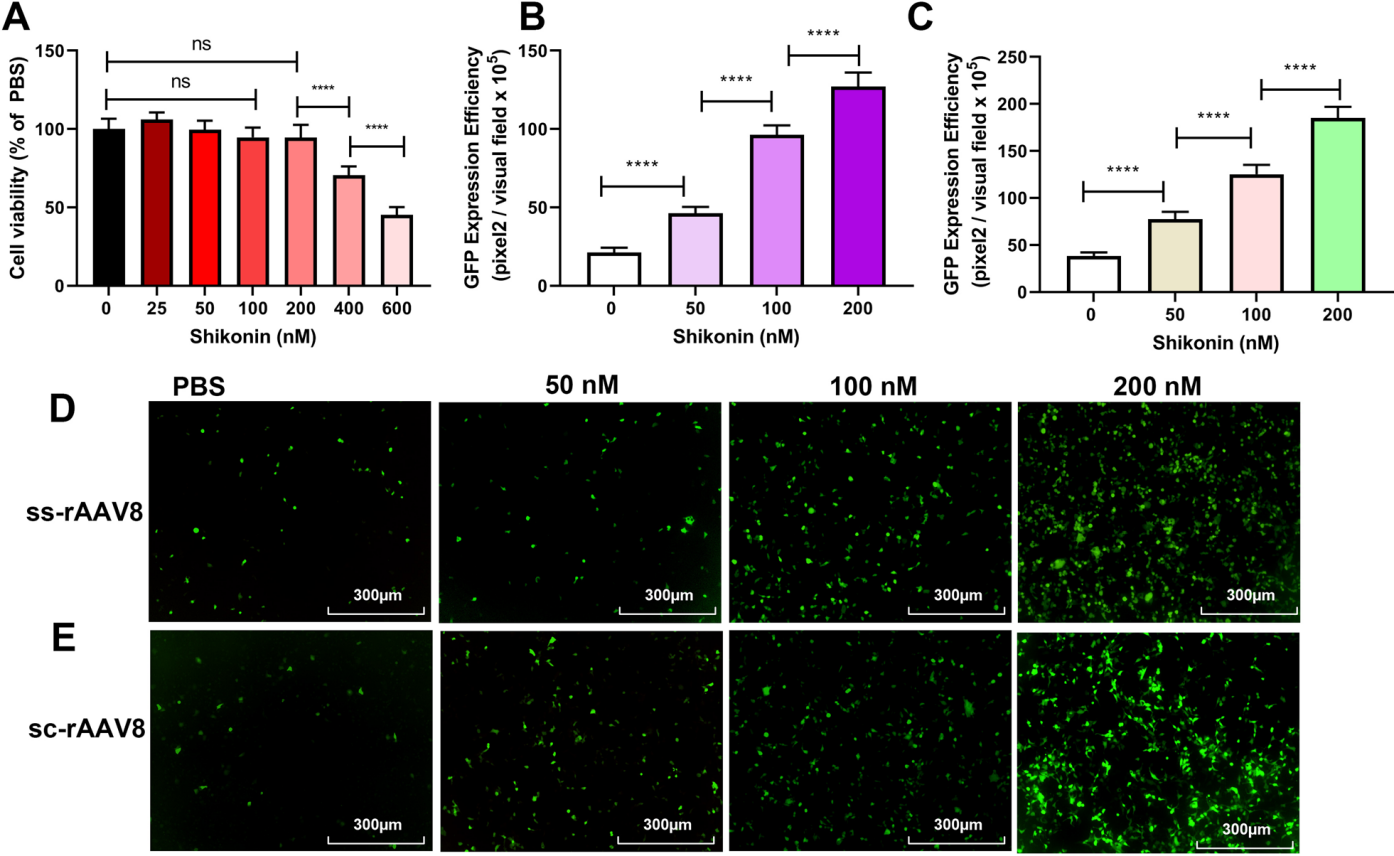
Shikonin pretreatment increased ssAAV8-GFP or scAAV8-GFP transduction in LO-2 cells. **A** Cell viability results of LO-2 exposed to 24-h shikonin treatment at different doses. Quantitation of transduction efficiency of ss-rAAV8 at 500 vg/cell (**B**) or sc-rAAV8 at 300 vg/cell (**C**) in LO-2 cells that treated with different doses of shikonin. The representative images of ss-rAAV8 (**D**) and sc-rAAV8- mediated (**E**) transgene expression in pretreated LO-2 were measured using fluorescence microscopy after the infection for 48 h. Data are represented by means ± SD (n = 3). ^ns^ *p* > 0.05; ****p < 0.0001

Considering that the liver is the ideal target for rAAV gene therapy and that the liver-tropism of rAAV8 vectors has been reported following intravenous administration, we hypothesized that rAAV8 transduction of hepatocytes could also be enhanced by shikonin. As expected, the transduction of LO-2 cells with both ss-rAAV8 and sc-rAAV8 was enhanced significantly (Fig. 2B–E), compared with the control, and the shikonin concentrations used had almost no effects on cell viability (Fig. 2A). These results confirmed the ability of shikonin to increase rAAV transduction, finding no AAV serotype and cell type dependence.

### Shikonin increases ss-rAAV8 transduction in vivo

**Fig. 3:**
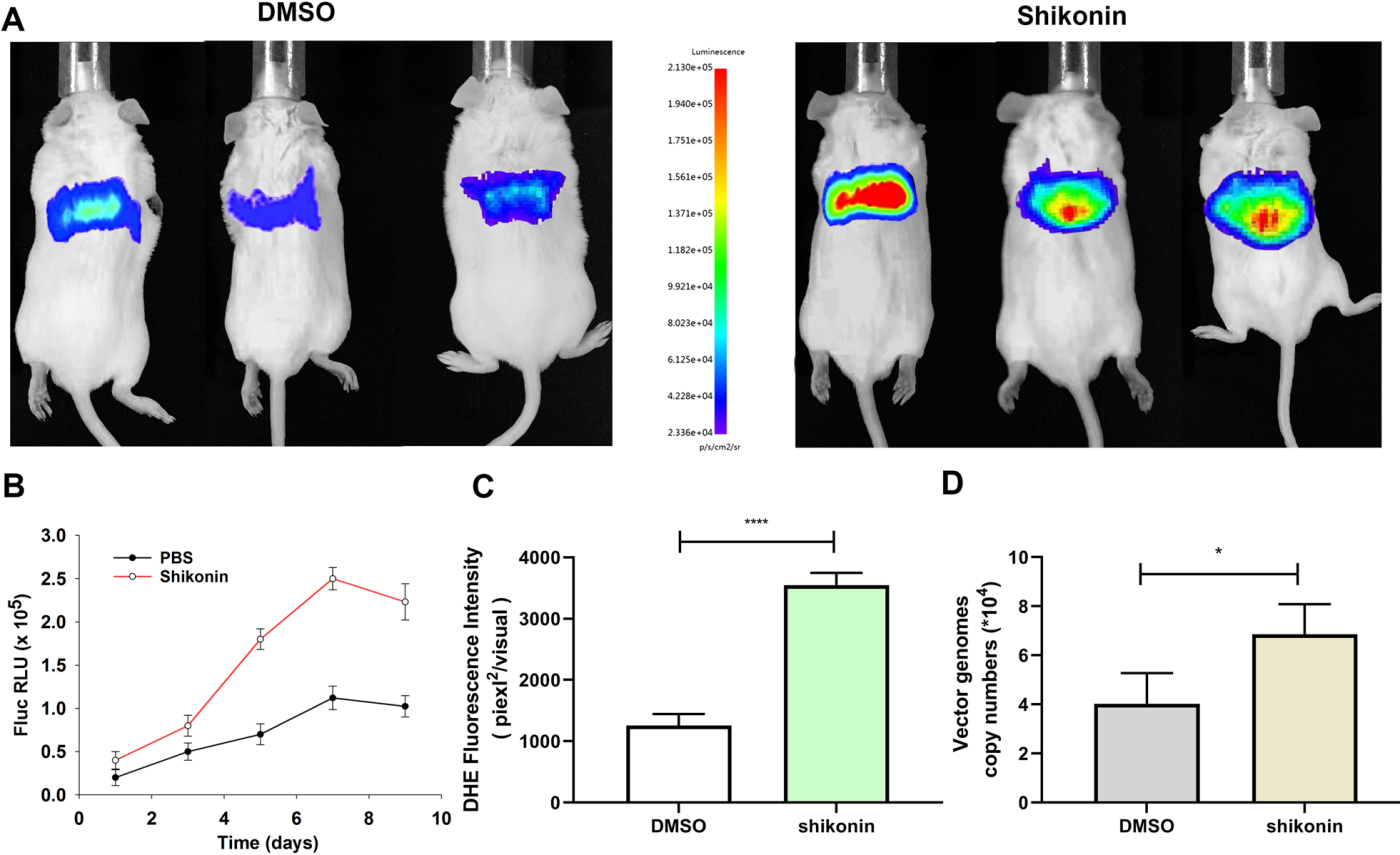
Shikonin increased ss-rAAV8 transduction in vivo. NSG mice treated with shikonin or DMSO were given an injection of ss-rAAV8-Fluc vectors via tail vein at 1 × 10^11^ vg/mouse, with six mice in each group. **A** 9 days after injection, whole-body bioluminescence imaging was performed on each NSG mouse. **B** Quantification on mouse bioluminescent signals 1/3/5/7/9 days following ss-rAAV8-Fluc treatment. **C** 3 days after ss-rAAV8-Fluc administration, ROS content was quantified in the liver by a fluorometer. **D** 100 ng total DNA from liver was employed to determine the number of vector genome copies in the liver 3 days post-transduction. All values represented are means ± SD (n = 3). *p < 0.05; ****p < 0.0001

Next, we investigated whether shikonin could increase ss-rAAV8 transduction in vivo. NSG male mice were injected intraperitoneally with shikonin (2 mg·kg^−1^·d^−1^) or an equal volume of DMSO solution for 3 days. On day 2, ss-rAAV8-Fluc (1 × 10^11^ viral genomes per mouse) was injected via the tail vein. Whole-body bioluminescence imaging was carried out 1, 3, 5, 7, and 9 days after rAAV8 administration. The results showed that shikonin treatment increased luciferase expression in the liver (Fig. 3A, B). On day 3 after rAAV8 administration, quantitative analysis of ROS generation in liver revealed that shikonin treatment increased the liver ROS level (Fig. 3C). We performed qPCR analysis on the viral genome copy number remained in liver tissues on day 3 after rAAV8 administration. The results suggested that shikonin treatment significantly increased the persistency of the viral genomes in mouse liver (Fig. 3D). The experiment suggested that shikonin exposure increased the transduction of rAAV8-Fluc in vivo, which may be due to the elevated ROS generation.

### ROS promotes rAAV2 transduction

**Fig. 4:**
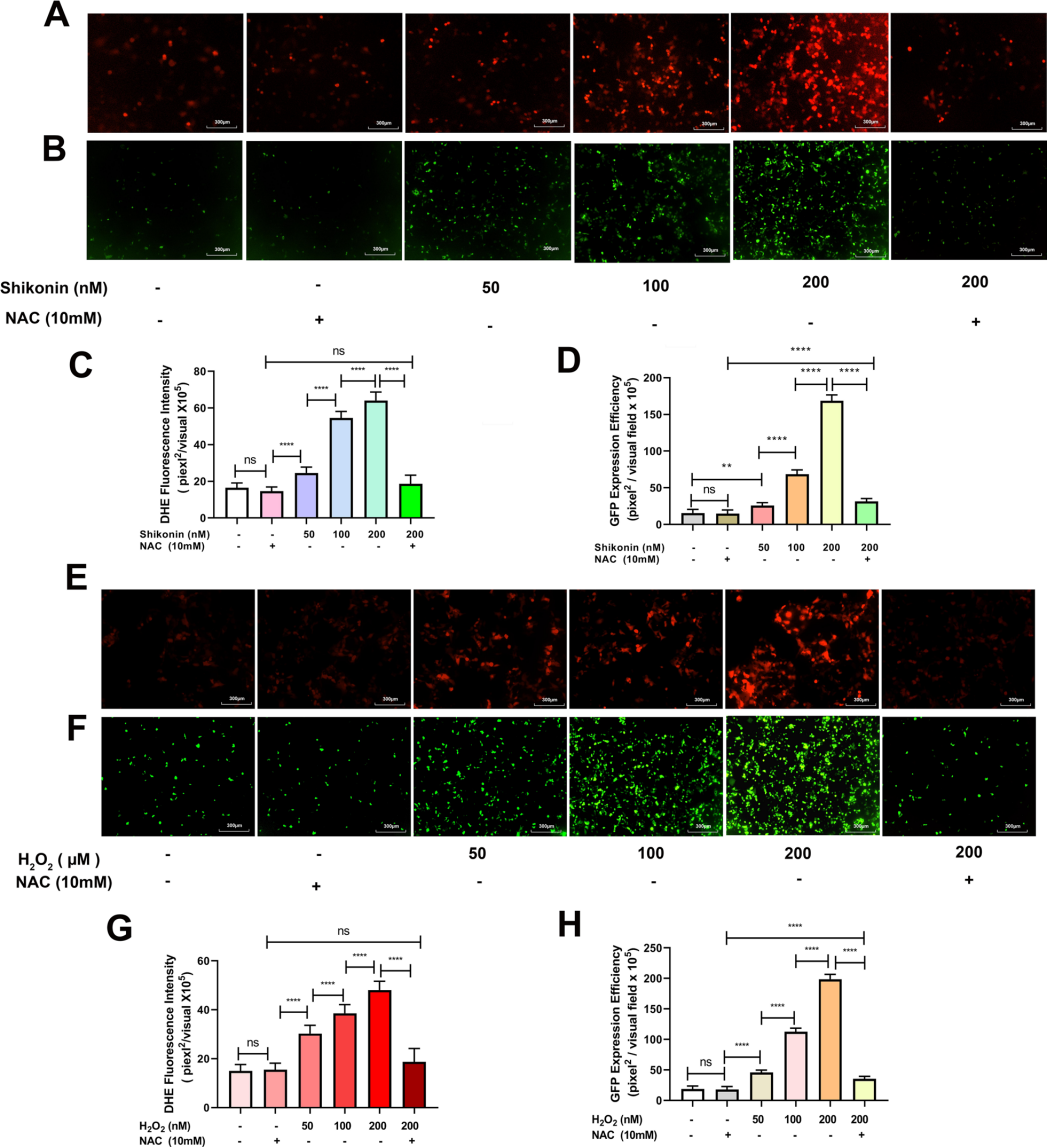
Depletion of ROS reduced rAAV2 transduction efficiency in Hela cells. Hela cell pretreated with shikonin, H_2_O_2_, or combined with NAC for 4 h, the medium was replaced, and rAAV2 was transduced. **A** Hela cells were treated with shikonin or combined with NAC for 4 h; DHE probes were used to measure ROS levels after 12-h pre-treatment. **B** Pretreated Hela cells with shikonin transduced with rAAV2-GFP vectors at 500 vg/cell. Transgene expression levels were detected under fluorescent microscopy 48 h after the transduction. **C** Statistical fluorescence intensity analysis with DHE fluorescent probe. **D** Statistical analyses of ss-rAAV2 transduction efficiency. **E** Hela cells treated with H_2_O_2_ or H_2_O_2_ combined with NAC for 4 h; ROS content was determined by DHE probes after 12 h of pre-treatment. **F** Pretreated Hela cells with H_2_O_2_ transduced with rAAV2-GFP vectors at 500 vg/cell. Fluorescent microscopy was conducted to obtain transgene levels 48 h following transduction. **G** Statistical fluorescence intensity analysis of DHE fluorescent probe. **H** Quantitative analyses of ss-rAAV2 transgene efficiency. All values indicated are means ± SD (n = 3). ^ns^ *p* > 0.05; **p < 0.01; ****p < 0.0001

To verify our hypothesis that shikonin enhances rAAV transduction through increased ROS generation, we treated Hela cells with shikonin, with or without N-acetyl-L-cysteine (NAC, 10 mM), a ROS scavenger. Similar as reported [1, 25], shikonin induced intracellular ROS production in a dose-dependent manner (Fig. 4A, C), and co-treatment with NAC could inhibit ROS production. Meanwhile, the increased expression of rAAV2 transgene GFP by shikonin was abolished after ROS scavenger NAC treatment (Fig. 4B, D).

H_2_O_2_ is a well-known ROS inducer and a classical reagent to study oxidative stress. Similar to shikonin, H_2_O_2_ also increased cellular ROS production and enhanced rAAV2 transduction in a dose-dependent way (Fig. 4E–H). The increased ROS level and rAAV2 transduction were abolished after NAC treatment, confirming that cellular ROS level was closely related to rAAV transduction efficiency.

### ROS improves rAAV2 intracellular trafficking

**Fig. 5:**
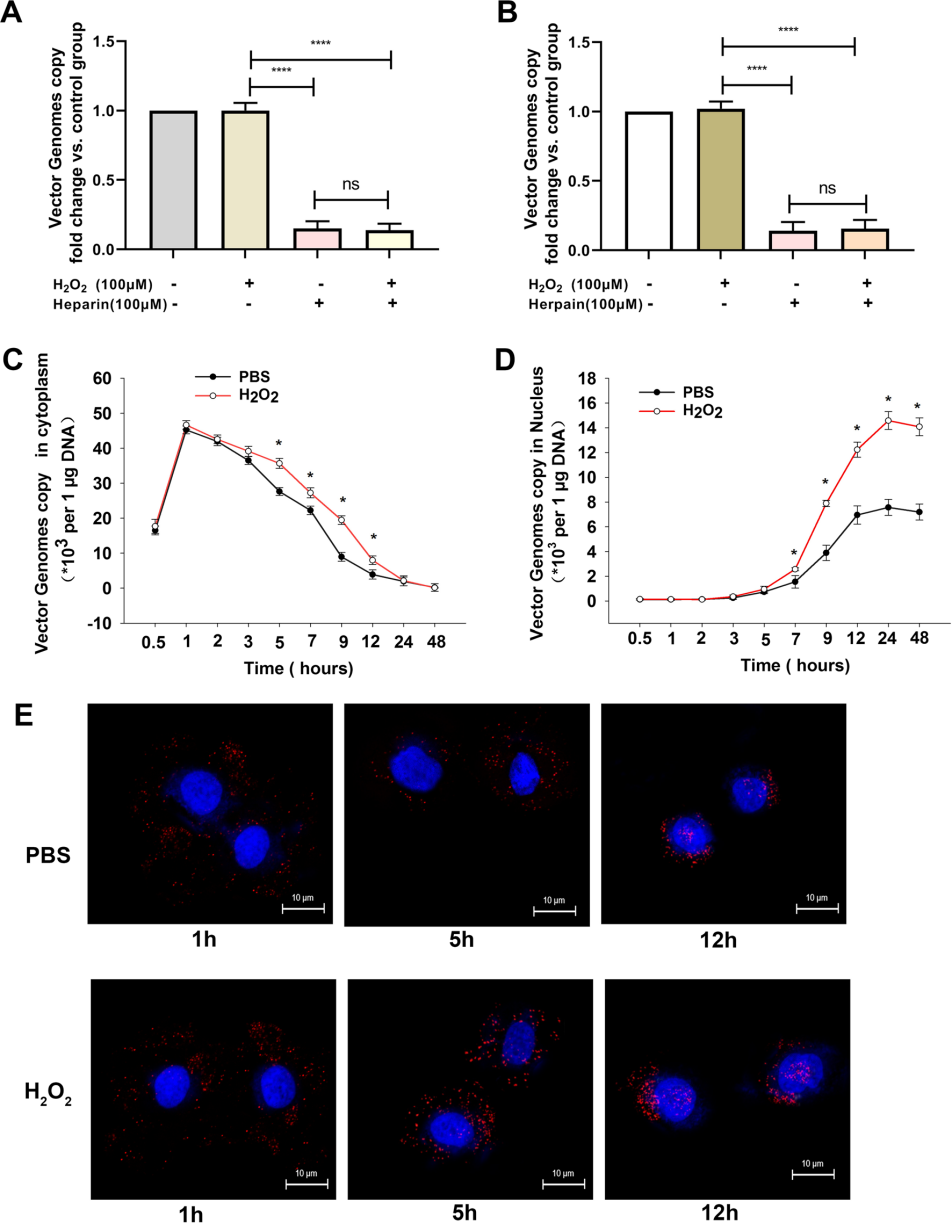
Effects of ROS on the intracellular distribution of rAAV2. **A** Binding assay was performed with vector-transfected Hela cells with/without heparin administration. **B** Internalization assay was conducted with vector-transfected Hela cells with/without heparin administration. **C** qPCR analysis of ss-rAAV2 vector genomes content in the cytoplasm after Hela cell pretreated with H_2_O_2_. **D** qPCR analysis of ss-rAAV2 vector genomes content in the nucleus after Hela cell pretreated with H_2_O_2_. **E** Subcellular localization of ss-rAAV2-TAMRA vectors after pre-treatment with H_2_O_2_. Hela Cells were fixed at 1 h, 5 h, or 12 h post-transduction. The localization of ss-rAAV2-TAMRA vectors was determined using confocal microscopy. TAMRA and DAPI signals are represented in red and blue, respectively. Data represent means ± SD (n = 3). ^ns^ *p* > 0.05; *p < 0.05 vs. PBS; ****p < 0.0001

To successfully transduce a target cell, rAAV must overcome some of the most prominent cellular roadblocks, such as rAAV cell binding and endocytosis, intracellular trafficking to the Golgi, nuclear import, and transgene expression. At first, we investigate the effect of H_2_O_2_ on rAAV2 binding and endocytosis. The results showed that heparin, the competitive inhibitor of AAV2 receptor heparan sulfate proteoglycans, strongly blocked cell binding and endocytosis of rAAV2; while H_2_O_2_ had no effects on rAAV2 binding and endocytosis (Fig. 5A, B). Next, we determined the time course of rAAV2 genome both in cytoplasm and nucleus by qPCR. According to the results, H_2_O_2_ treatment improved the intracellular trafficking efficiency (Fig. 5C, D), and the vector genome copy numbers were increased both in the cytoplasm and nucleus, with significant changes observed 5–7 h after transduction. To confirm that the facilitated intracellular trafficking of rAAV2 vectors occurs at the virion level, we infected Hela cells with fluorescently-labeled rAAV2. At 5 h after transduction, more perinuclear rAAV2 virion accumulation was found after H_2_O_2_ administration, and most of the viruses were distributed around or within the nucleus at 12 h after transduction (Fig. 5E). These results suggested that H_2_O_2_ could increase the rAAV intracellular trafficking efficiency in the cytoplasm. Therefore, we focused on the effect of H_2_O_2_ on rAAV2 transport in the cytoplasm in the following experiment.

### ROS facilitates rAAV2 escape from late endosome

**Fig. 6:**
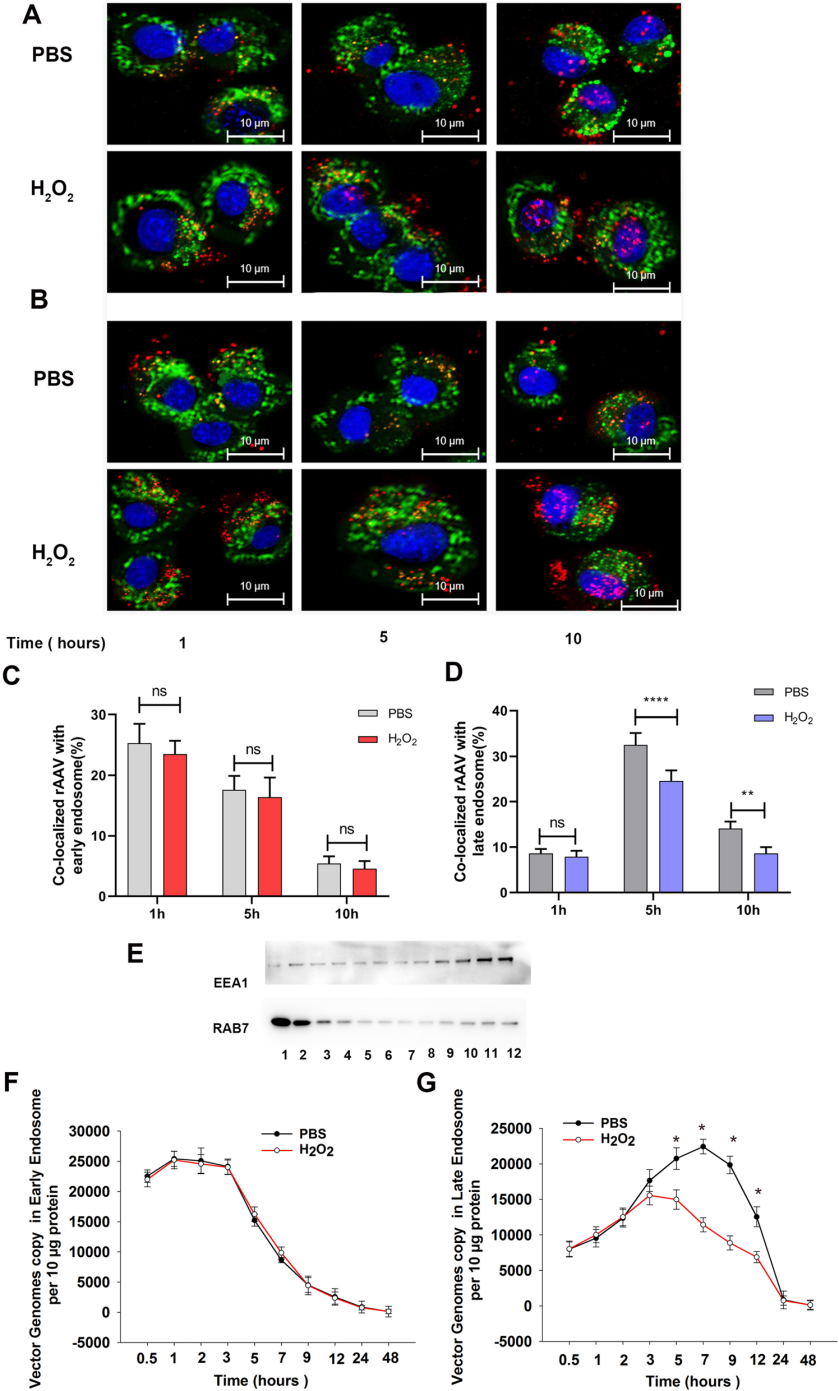
Effects of ROS on the intracellular distribution of rAAV2. **A** Localization of rAAV2-TAMRA in the early endosome when Hela cells were pretreated with H_2_O_2_ (100 nM) or without for 4 h and infected with rAAV2 at 2000 vg/cell at 37 °C. Early endosomes were measured using anti-EEA1 mAb after cell fixation at 1, 5, and 10 h post-infection. (Blue is the nucleus; Green is the early endosome; Red is rAAV2-TAMRA). **B** Localization of rAAV2-TAMRA in the late endosome when Hela cells were pretreated with H_2_O_2_ (100 nM) or without for 4 h and later infected with rAAV2 at 2000 vg/cell at 37 °C. Late endosomes were measured using anti-RAB7 mAb after cell fixation at 1, 5, and 10 h post-infection (Blue is the nucleus; Green is the late endosome; Red is rAAV2-TAMRA). **C** Percentage of rAAV co-localized with early endosome. **D** Percentage of rAAV co-localized with a late endosome. **E** WB assay on density-gradient fractions (#1–12; n = 12) collected in HeLa cells. The early endosome and late endosome markers EEA1 and RAB7 were detected by blotting. EEA1 is enriched in low-density fractions #10–12; RAB7 is enriched in high-density fractions #1–3. qPCR analysis of rAAV2 genomic in early endosomes (**F** and late endosomes **G** within 48 h of rAAV transduction with cells being pretreated with H_2_O_2_ for 4 h. The results are represented by means ± SD (n = 3). ^ns^ *p* > 0.05; *p < 0.05 vs. PBS; ** *p* < 0.01 ****p < 0.0001

After endocytosis, rAAV traffics in the cytoplasm following the early endosome to late endosome to trans-Golgi network pathway. Although Golgi localization is critical for rAAV transduction, rAAV must escape into the cytosol before it can be imported into the nucleus. The gradually decreased pH from the early endosome to the late endosome induces rAAV to undergo significant conformational changes, which is suggested as a critical step for the escape of rAAV into the cytosol. Otherwise, the rAAV containing late endosomes will be fused with lysosomes responsible for degradation. To confirm the hypothesis that ROS facilitates rAAV’s escape from late endosome into the cytosol, we tracked rAAV2 cytoplasm trafficking with fluorescently labeled rAAV2 particles and endosome immunofluorescence staining (Fig. 6A, B). After the early endosome and late endosome were isolated by density gradient centrifugation, then the rAAV2 particles time course in the early endosome and late endosome was analyzed by qPCR (Fig. 6C, D, F, and G). The early endosome labeled with EEA1 was enriched in low-density fractions, and the late endosome labeled with RAB7 was enriched in high-density fractions (Fig. 6E). H_2_O_2_ treatment had little effect on rAAV2 distribution in the early endosome; however, decreased rAAV2 distribution in the late endosome was observed, especially at 5 h after transduction. These results indicated that ROS facilitated the timely escape of rAAV2 from late endosomes, which is beneficial for preventing proteasome-mediated degradation in the lysosome.

### ROS promotes rAAV2 release from late endosomes by up-regulating cathepsin B and L expression

**Fig. 7:**
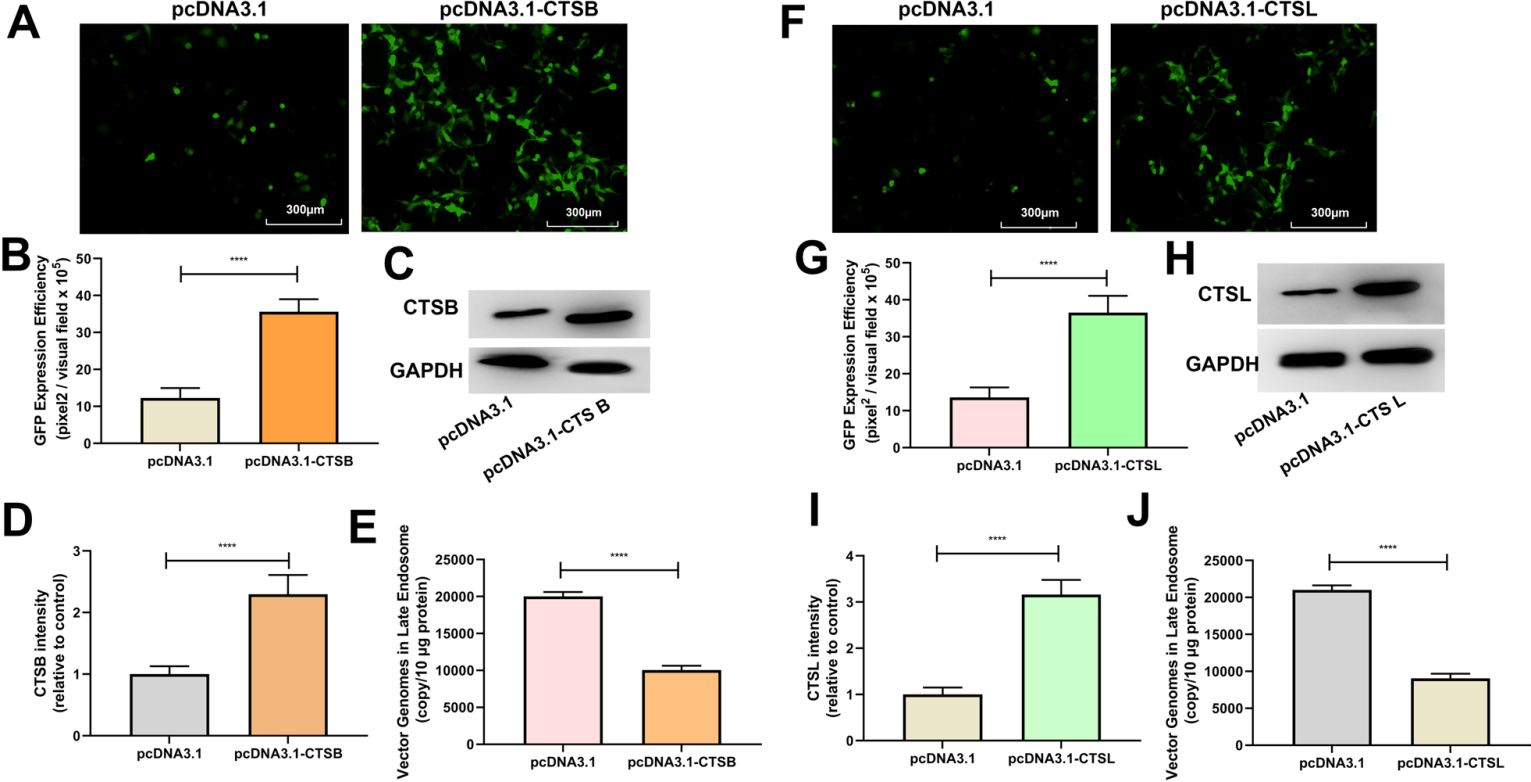
Effects of cathepsins B or cathepsins L overexpression on rAAV2 transduction. pcDNA3.1-CTSB, pcDNA3.1-CTSL, or pcDNA3.1 plasmid was transfected into HeLa cells for 24 h, then transduced with rAAV2-GFP at 500 vg/cell. **A** The representative images of pcDNA3.1 or pcDNA3.1- CTSB-transfected and rAAV2-GFP-transduced HeLa cells for 48 h. **B** Quantification of rAAV2 transduction efficiency after pcDNA3.1 or pcDNA3.1-CTSB pretreatment in cells. **C** After treatment for 24 h, the CTSB level was measured through WB assay. **D** Quantitative analysis of CTSB expression. **E** Quantitative analysis of vector genomes in late endosomes when rAAV-GFP transduced the pretreated Hela cells for 7 h. **F** The representative images of pcDNA3.1 or pcDNA3.1-CTSL-transfected and then rAAV2-GFP-transduced HeLa cells for 48 h. **G** Quantitation of transduction efficiency of rAAV2 after cell pretreated with pcDNA3.1 or pcDNA3.1-CTSL. **H** After treatment for 24 h, the CTSL level was measured through WB assay. **I** Quantitative analysis of CTSB expression. **J** Quantitative analysis of vector genomes in late endosomes when rAAV-GFP transduced pretreated Hela cells for 7 h. In addition, all data are represented by means ± SD (n = 3). ****p < 0.0001

**Fig. 8:**
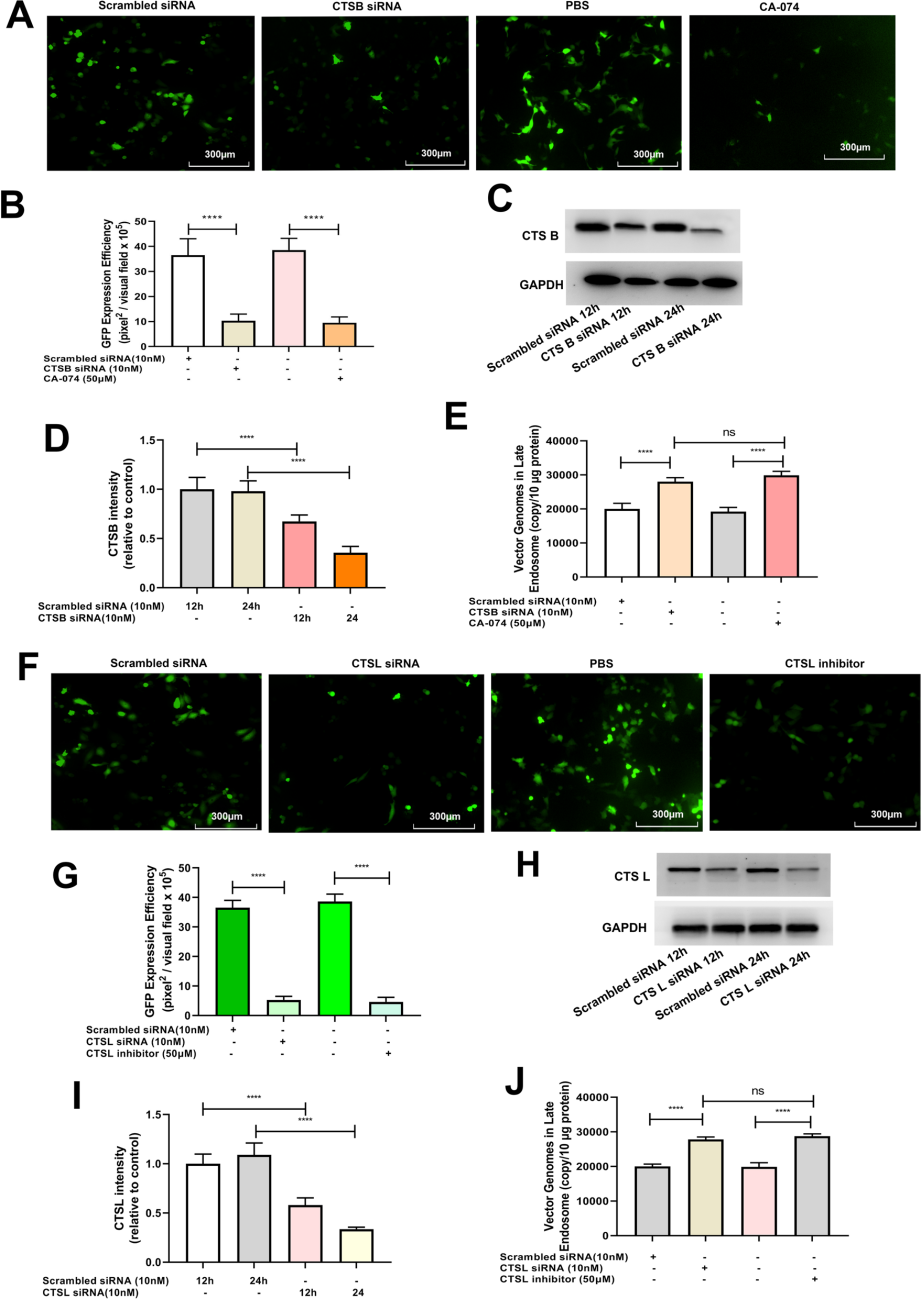
Effects of cathepsins B or cathepsins L silencing on rAAV2 transduction. Hela cells were pretreated with CA-074, cathepsins L inhibitor for 4 h or transfected with 10 nM CTSB siRNA, CTSL siRNA or scrambled siRNA for 24 h, then transduced with rAAV2-GFP at 1000 vg/cell. **A** The representative images of Hela cells transfected with scrambled siRNA, CTSB siRNA or pretreated with CA-074 and then transduced with rAAV2-GFP for 48 h. **B** Quantification of rAAV2 transduction efficiency after CTSB siRNA or CA-074 pretreatment. **C** After treatment, the CTSB level was measured through WB assay. **D** Quantitative analysis of CTSB expression. **E** Quantitative analysis of vector genomes in late endosomes when rAAV2-GFP transduced Hela cells for 7 h, which was pretreated with CTSB siRNA or CA-074. **F** The representative images of Hela cells transfected with scrambled siRNA, CTSL siRNA, or pretreated with CTSL inhibitor and then transduced with rAAV2-GFP for 48 h. **G** Quantitation of transduction efficiency of rAAV2 after the cell was pretreated with CTSL siRNA or CTSL inhibitor. **H** After treatment, the CTSL level was measured through WB assay. **I** Quantitative analysis of CTSL expression. **J** Quantitative analysis of vector genomes in late endosomes when rAAV-GFP transduced Hela cells for 7 h pretreated with CTSL siRNA or cathepsins L inhibitor. All values shown are means ± SD (n = 3). ^ns^ *p* > 0.05; ****p < 0.0001

After endocytosis, the endosomes containing rAAV can presumably follow different trafficking ways, resulting in successful transduction or causing degradation. The difference in the kinetics of H_2_O_2_’s effect on the endosomal trafficking of rAAV2 indicates a role for ROS in the transduction of rAAV2. A series of pH-dependent structure changes during endosomal trafficking are necessary steps for transduction. It has been suggested that endosomal proteases cathepsin B and L’s proteolytic processing of rAAVs at low pH is also responsible for the conformational changes of rAAVs [21]. We proposed that these two endosomal proteases may play a role in rAAV escape from late endosomes. As expected, the overexpression of cathepsins B promoted rAAV2 transduction (Fig. 7A–D), and enhanced rAAV2 escape from late endosomes (Fig. 7E). Similarly, the overexpression of cathepsins L also promoted rAAV2 transduction (Fig. 7F–I), and enhanced rAAV2 escape (Fig. 7J). In addition, when cathepsins B or L was silenced by siRNA (Fig. 8C, D, H, and I), the transduction efficiency of rAAV2 was decreased to a level, which was almost similar to that treated with cathepsin B inhibitor, CA-047, or cathepsin L inhibitor (Fig. 8A, B, F, and G). The degree of rAAV2 vector escape from late endosomes after cathepsin B, or L silence was lower than that of the control group (Fig. 8E, J).

**Fig. 9:**
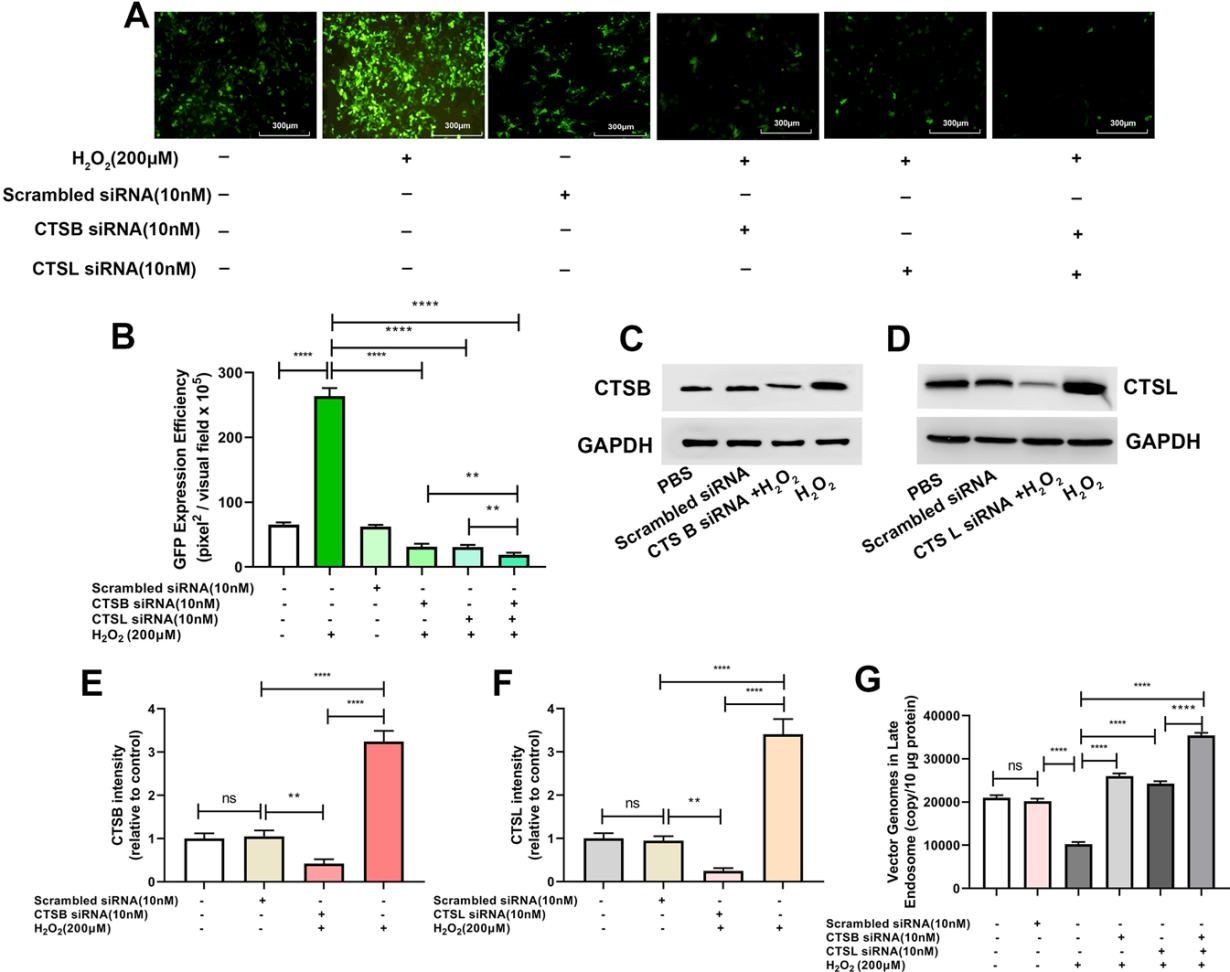
Role of ROS in rAAV2 transduction by regulating cathepsins B and L. Hela cells were transfected with 10 nM CTSB siRNA, CTSL siRNA, or scrambled siRNA for 24 h, and then pretreated with H_2_O_2_ for 4 h. The pretreated Hela cells were subject to transfection using rAAV2-GFP at 1000 vg/cell. **A** The representative images of Hela cells transfected with CTSL siRNA, CTSB siRNA and pretreated with H_2_O_2_ and then transduced with rAAV2-GFP for 48 h. **B** Quantification of rAAV2 transduction efficiency after CTSB siRNA, CTSL siRNA and H_2_O_2_ pretreatment. **C** After the treatment, the CTSB level was determined using WB assay. **D** After the treatment, CTSL expression was measured through WB assay. **E** Quantitative analysis of CTSB expression. **F** Quantitative analysis of CTSL expression. **G** Quantification of rAAV2 in late endosomes when pretreated Hela cells were transduced with rAAV2-GFP for 7 h. Data are represented by means ± SD (n = 3). ^ns^ *p* > 0.05; ****p < 0.0001

Then, we set out to identify whether H_2_O_2_ treatment influenced cathepsin B or L expression. Expression of both cathepsins B and L was increased after H_2_O_2_ exposure (Fig. 9C–F). Meanwhile, the rAAV2 transgene expression was enhanced (Fig. 9A, B). On the contrary, the effects of H_2_O_2_ exposure disappeared after cathepsins B and L were silenced by siRNA. Similarly, the enhancement of rAAV2 escape from late endosomes by H_2_O_2_ was also abolished after cathepsins B and L silence (Fig. 9G). These results demonstrated that H_2_O_2_ promoted rAAV2 escape from late endosomes by up-regulating the expression of cathepsins B or L.

**Fig. 10:**
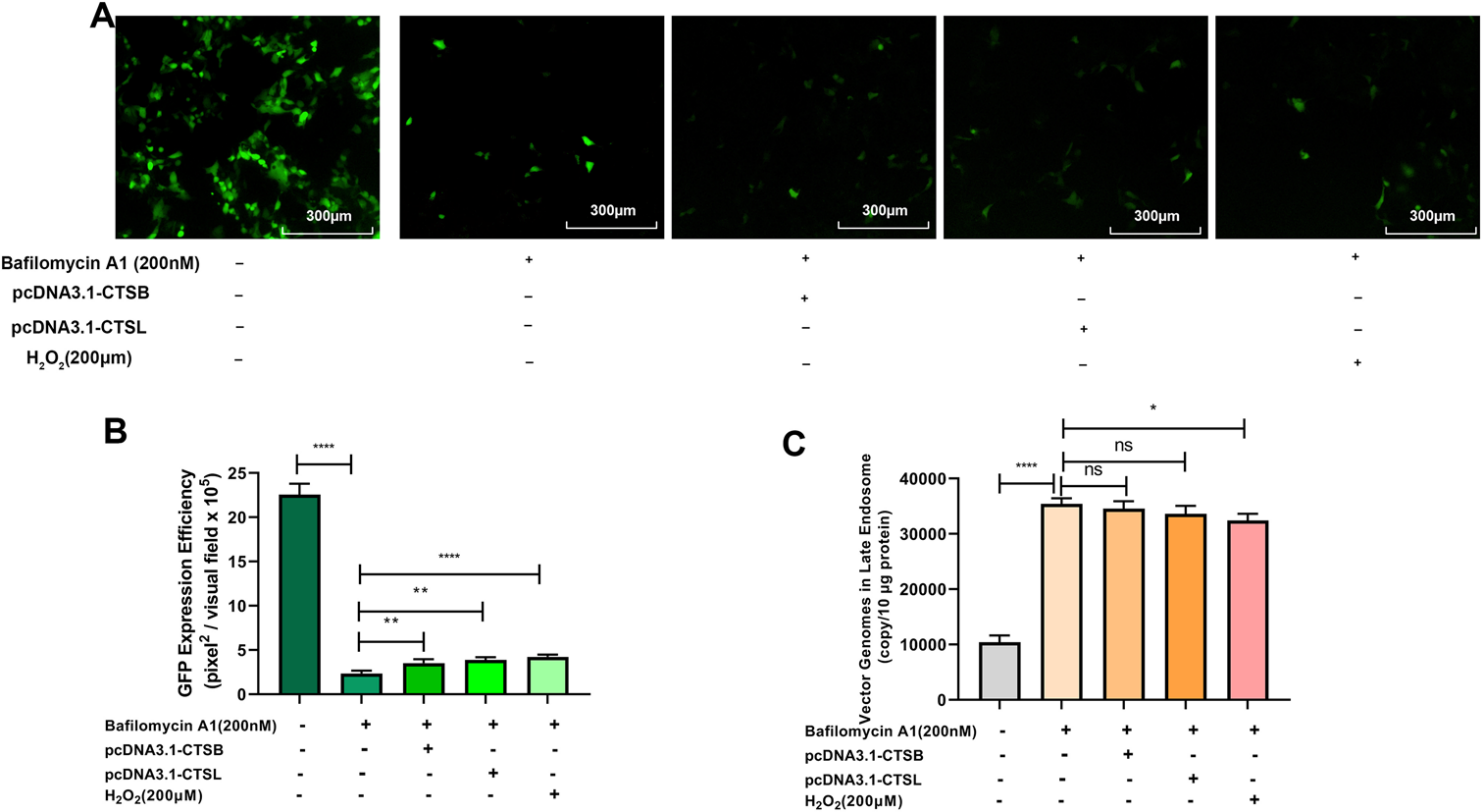
Effects of endosome acidification on cathepsins B, cathepsins L, or ROS-enhanced rAAV2 transduction. pcDNA3.1-CTSB, pcDNA3.1-CTSL was transfected into HeLa cells for 24 h or pretreated with bafilomycin A1, and then H_2_O_2_ transduced with rAAV2-GFP at 1000 vg/cell. **A** Effects of acidic endosomal environment on ROS-promoted rAAV transgene expression. **B** GFP level was determined 48 h after infection. **C** Quantification of rAAV2 in late endosomes when rAAV2-GFP was transfected into HeLa cells for a 7-h period. Data are indicated by means ± SD (n = 3). ^ns^ *p* > 0.05; *p < 0.05; **p < 0.01; ****p < 0.0001

rAAVs require acidification during cellular trafficking for successful transduction. Endosome acidification can not only induce capsid modification to facilitate the escape from endosome [26, 27], but also can trigger proteases activation, including cathepsin B and L. Once Bafilomycin A1 inhibited endosome acidification, the enhanced effects of rAAV2 transduction by overexpression of cathepsins B or L, or H_2_O_2_ exposure, were abolished (Fig. 10A, B), partly because more rAAV2 vectors were trapped in late endosomes (Fig. 10C). Moreover, the results also demonstrate that inefficient capsid trafficking through the endosome/Golgi networks is an obstacle to successful rAAV gene delivery.

## Discussion

**Fig. 11:**
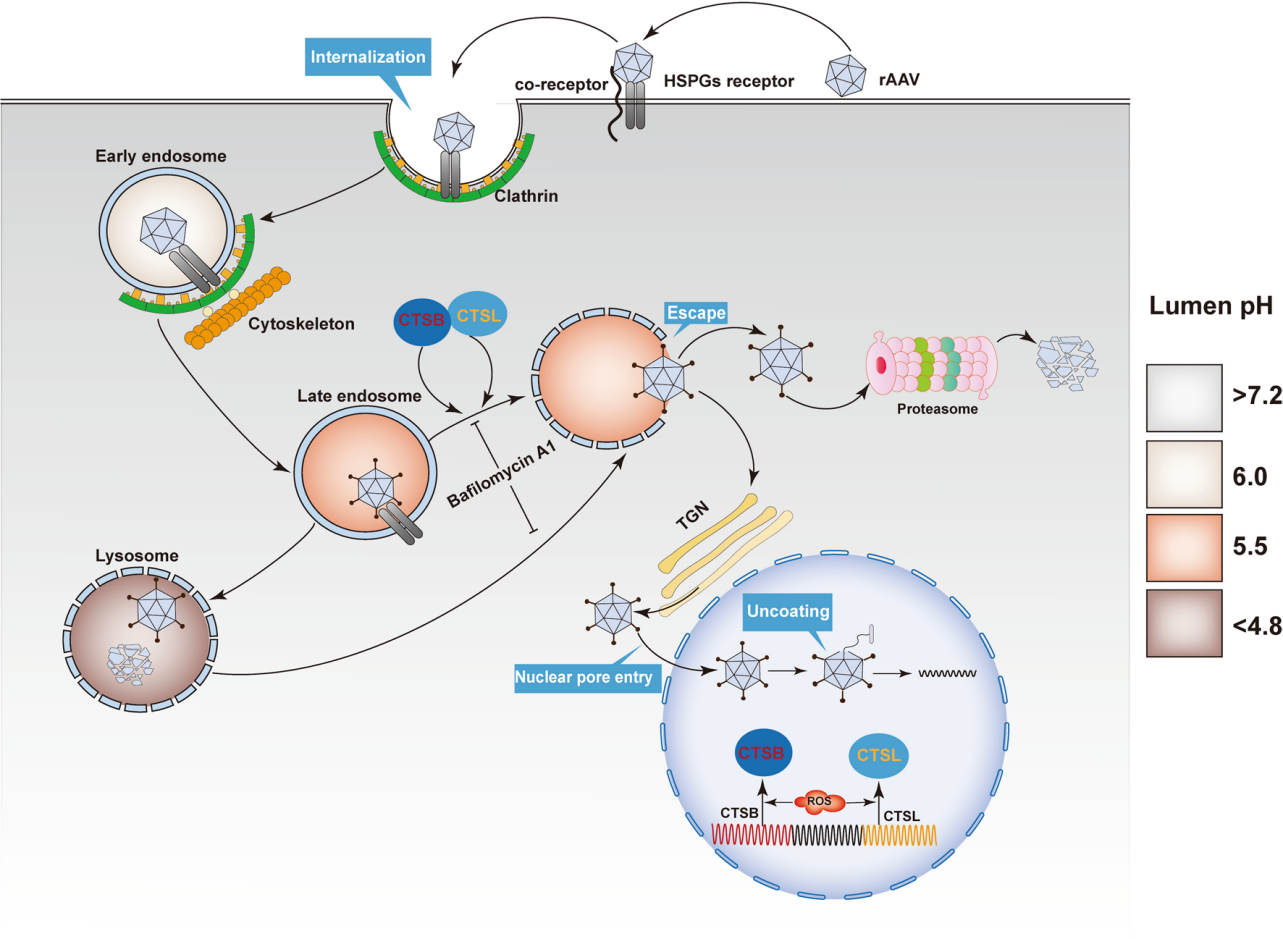
A model that ROS can facilitate rAAV2 escape from late endosomes to promote rAAV2 transduction. After binding to the HSPGs/co-receptor complex, rAAV enters target cells through clathrin-mediated endocytosis. When a particle entered the endosome, due to the acidic endosomal environment, cathepsins B was combined with L, and the capsids were cleaved. VP1/VP2 region underwent a conformational change, and phospholipase A2 (PLA2) domain exposure (spikes) promoted the release of cytoplasm from the Golgi apparatus. After processing particles, they were released from endosome to trans-Golgi network (TGN) via the retrograde transport pathway. After nuclear import, intact capsids were mobilized in the nucleoplasm, followed by partial uncoating-mediated genome release

ROS are highly reactive molecules. To a large extent, their generation site will thus identify their site of action. Several recent studies have shown that endosomes are specialized compartments for ROS production and redox signaling as a response to virus infection. Virus-driven endosomal ROS do not eliminate viruses in a way similar to that of bacteria and fungi. By contrast, endosomal ROS facilitates rather than inhibits viral infection. Intriguingly, ROS generators, including shikonin and H_2_O_2_, have also been reported to interfere with cellular ROS production and enhance rAAV transduction. In the current study, we observed that ROS treatment increased rAAV escape from late endosomes, which redirects the intracellular trafficking of rAAV particles from a pathway of early endosomes to late endosomes. Lysosomes, toward a pathway of early endosomes to late endosomes, then trans-Golgi network (Fig. 11). More rAAV particles have the chance for nucleus entry preventing rAAV degradation in lysosomes. Therefore, enhanced recovery of rAAV genomes from the nuclear fraction was found.

After internalization, rAAV must travel to the Golgi for successful transduction [28–31]. During its trafficking to the Golgi, the rAAV particles undergo significant conformational changes triggered by the endosome [1]. The acidic environment is critical in unique N-terminal externalization in minor capsid VP1 of AAV, which possesses one phospholipase A2 (PLA2) domain, promoting the virus escape from the endosome/lysosomal pathway to the nucleus. Although endosomal acidification is essential during AAV2 processing, endosomal acidification alone is not sufficient to externalize VP1/2 [32]. Bassel el at. showed that cathepsin B and L proteins bound to and cleave the complete AAV2/AAV8 particles *in-vitro*, and they exerted vital effects on the efficient transduction of AAV2/AAV8 [27]. Our results demonstrated that the overexpression of cathepsins B and L could significantly increase rAAV2 transduction, while silencing cathepsins B and L significantly suppressed rAAV2 transduction, indicating the role of cathepsins B and L in promoting rAAV2 transduction (Figs. 7 and 8). The overexpression of cathepsins B and L significantly reduced the number of viral genomes in late endosomes, while silencing cathepsins B and L significantly increased the number of viral genomes in late endosomes, implying their roles in promoting rAAV2 escape from the endosome.

Another important observation is that ROS affects the expression and activities of cathepsin B and L (Fig. 11). Previous researches has indicated that trafficking to the Golgi is a vital step in rAAV transduction. However, rAAV must also undergo conformational changes during endosomal trafficking [33]. Infections by the Ebola virus, SARS-CoV, SARS-CoV-2, and murine leukemia virus require the cleavage of their capsid by cathepsin B or L protease to activate their membrane fusion capability [34], Akache et al. also suggested that cathepsin-mediated cleavage could prime AAV capsids for subsequent nuclear uncoating [21]. In this study, ROS treatment appears to increase the expression of both cathepsin B and L and accelerate the rAAV transit in the late endosome stage. These observations appear to be inconsistent with the earlier studies revealing that rAAV transport via late endosomes/lysosomes is highly productive compared to early/recycling endosomes [35]. However, several groups demonstrated that for successful transduction, rAAV must be transported to the Golgi [36, 37]. For example, the treatment with brefeldin A7 or Golgicide A disrupts the Golgi apparatus structure and abolishes rAAV2 transduction [37]. In NIH3T3 cells, rAAV2 successfully binds to the cells and is efficiently endocytosed, while the transduction is very low since the transport of rAAV2 to the Golgi is severely impaired [37]. The overall improved effects of ROS on rAAV transduction are further supported by (1) the increased recovery of vector genome copy numbers from nucleus at late time intervals after rAAV transduction and (2) the enhanced transgene expression.

A key question is whether such studies have the potential for translational impact in rAAV-mediated gene therapy. Additionally, the tragic death of three children in a clinical trial for treating X-linked myotubular myopathy with an rAAV vector has sounded the alarm for us again. It is critical to develop better protocols for rAAV gene therapy to achieve therapeutic goals with lower vector doses. In this regard, numerous small molecular drugs have been evaluated for their potential applications. However, the present study can constitute an important step forward in understanding the molecular mechanism that influences rAAV intracellular trafficking and highlights the potential for using small molecular drugs, such as shikonin, aiming to augment rAAV transduction.

## Data Availability

All data generated or analyzed during this study are included in this published article.

## Abbreviations

rAAV: Recombinant adeno-associated virus
ssrAAV: Single-stranded recombinant AAV
scrAAV: Self-complementary recombinant adeno-associated virus
CTSB: Cathepsins B
CTSL: Cathepsins L
H_2_O_2_: Hydrogen peroxide
ROS: Reactive oxygen species
