# Supplementary material for: Reactive oxygen species enhance rAAV transduction by promoting its escape from late endosomes
Source: Virol J. 2023 Jan 7;20:2. doi: 10.1186/s12985-023-01964-w (PMC9825130; doi:10.1186/s12985-023-01964-w)

Supporting information

Western blot origin image

Figure 6 C

EEA1

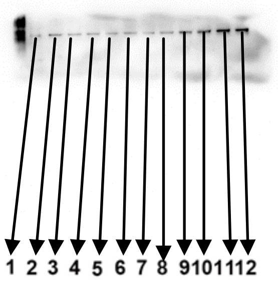

RAB7

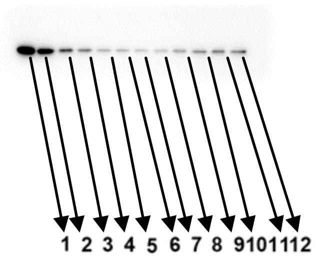

EEA1 replicate1

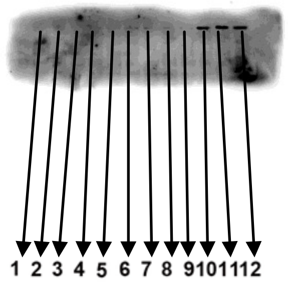

RAB7 replicate1

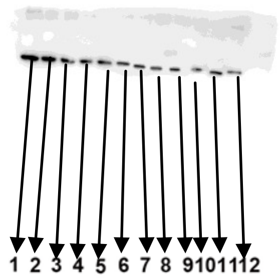

EEA1 replicate2

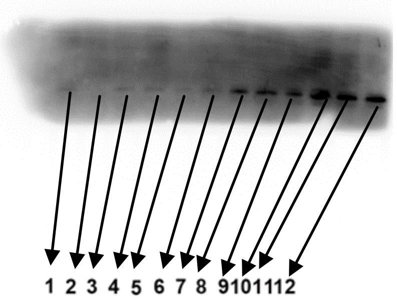

RAB7 replicate2

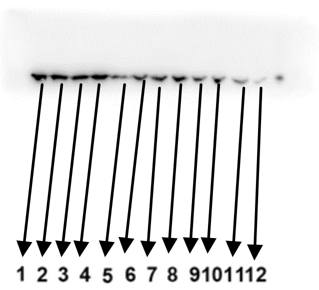

Figure7C

CTSB CTSB replicate1 CTSB replicate 2 CTSB replicate3

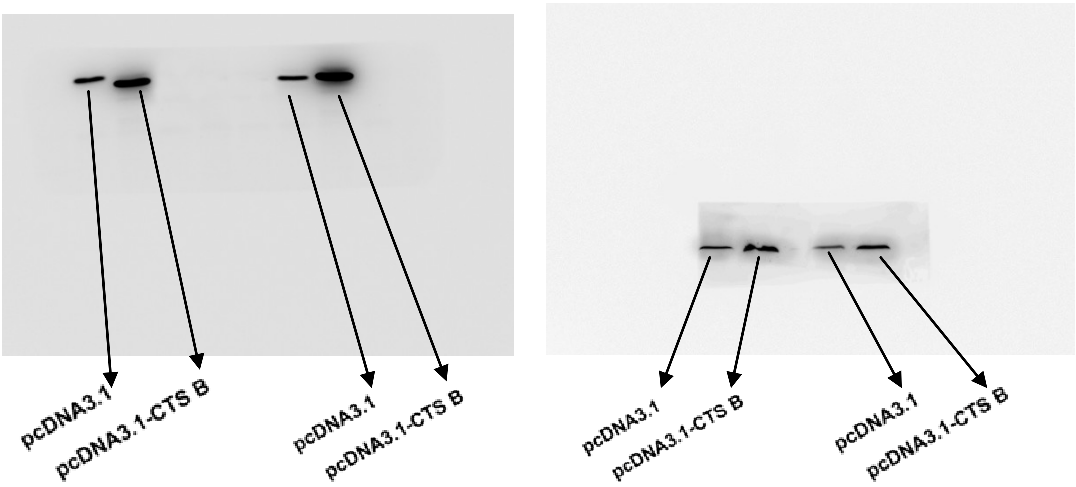

GAPDH GAPDH replicate 1 GAPDH replicate2 GAPDH replicate3

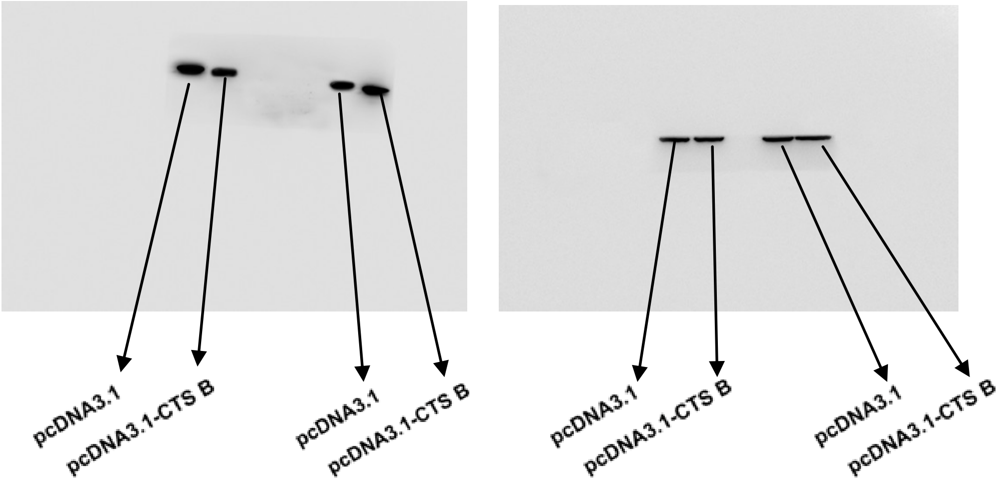

Figure 7H

CTSL CTSL replicate 1 CTSL replicate 2 CTSB replicate 3

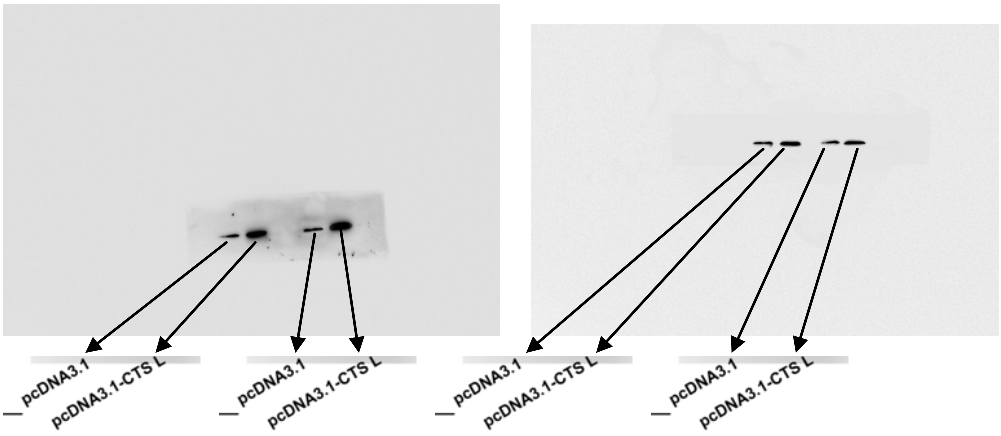

GAPDH **GAPDH replicate1**

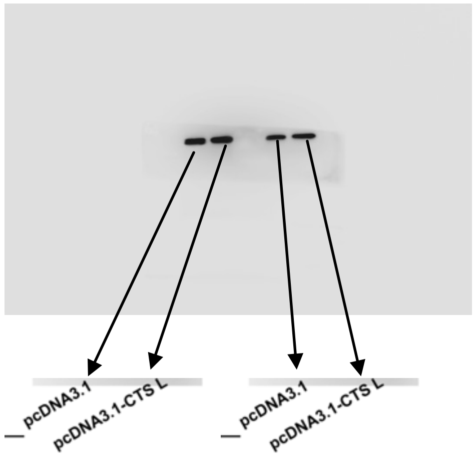

GAPDH replicate2 **GAPDH replicate3**

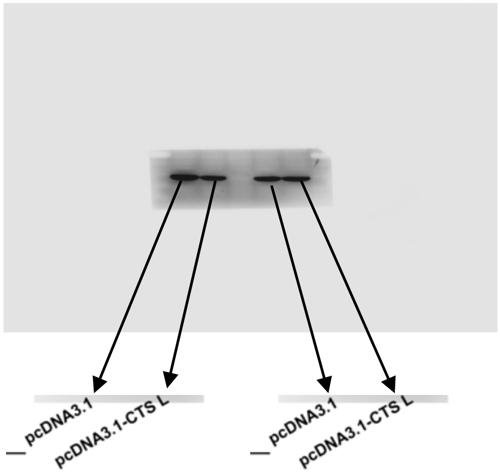

Figure 8 C

CTSB siRNA

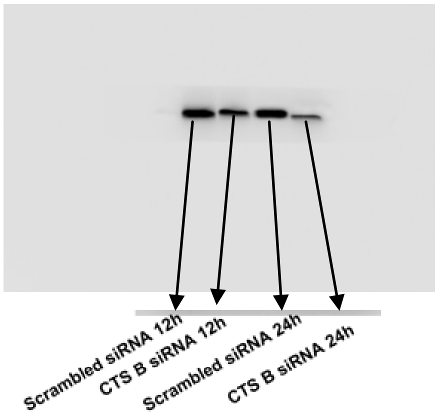

GAPDH

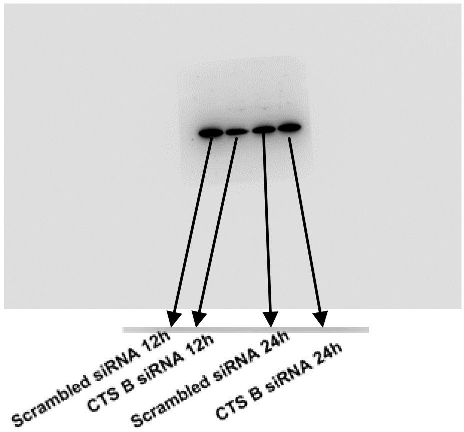

CTSB siRNA replicate 1

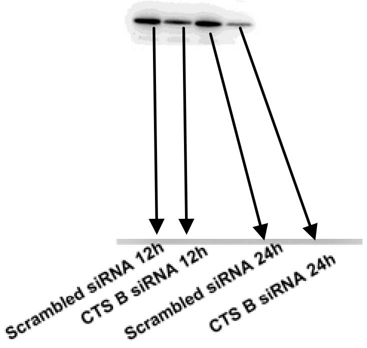

GAPDH replicate 1

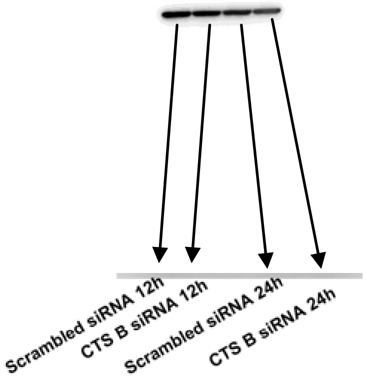

CTSB siRNA replicate 2

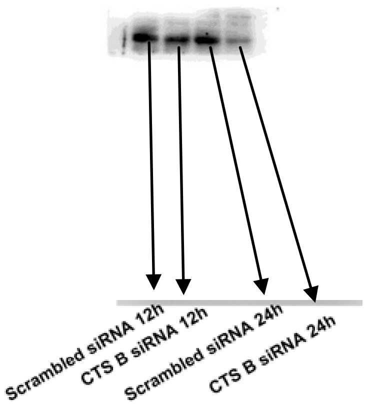

GAPDH replicate 2

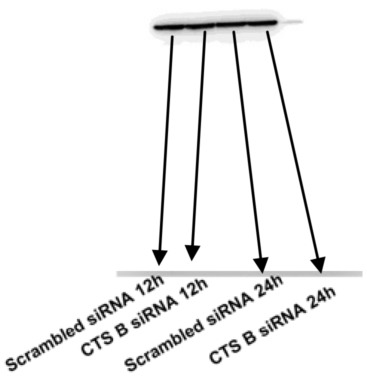

Figure 8 H

CTSL siRNA

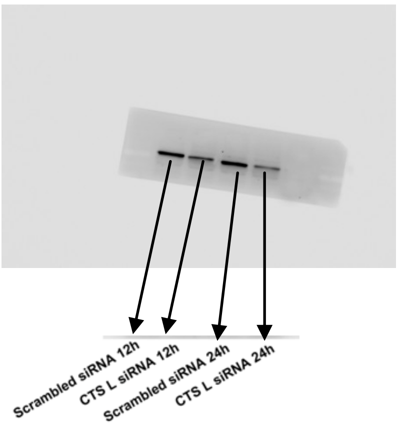

GAPDH

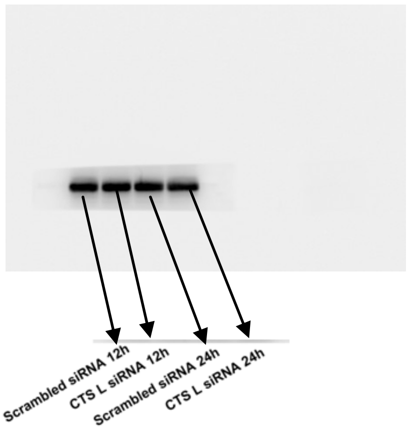

CTSL siRNA replicate 1

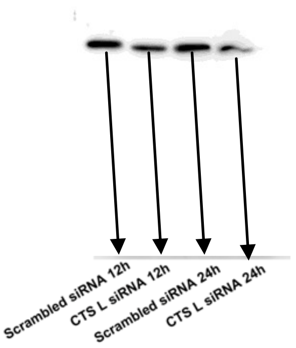

GAPDH replicate 1

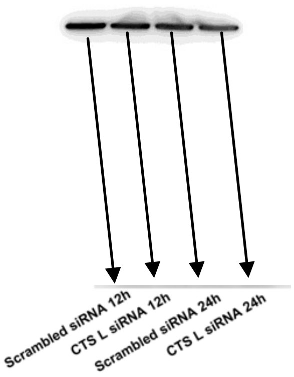

CTSL siRNA replicate 2

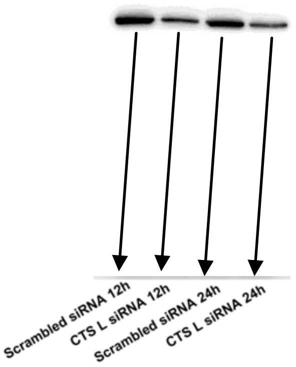

GAPDH replicate 2

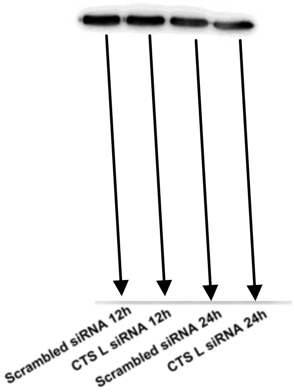

Figure 9 C

CTSB GAPDH

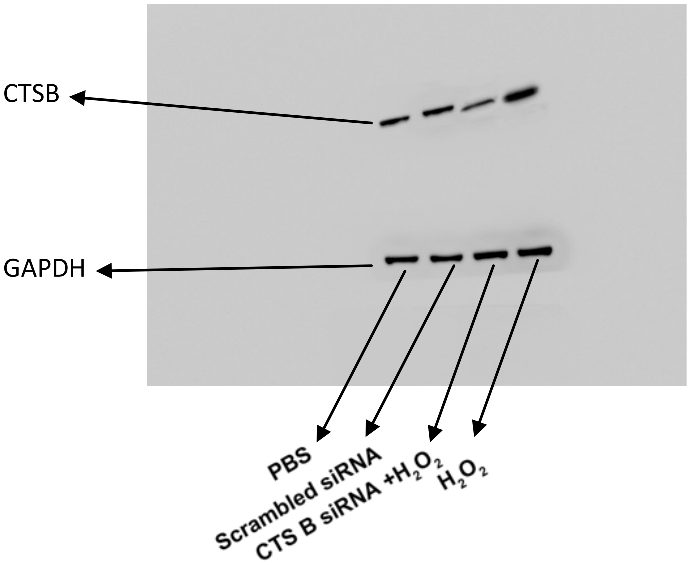

CTSB replicate 1

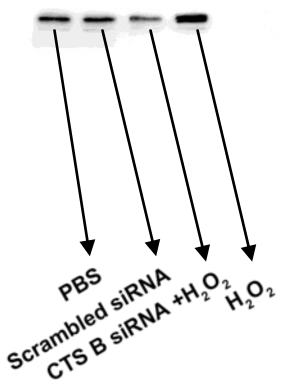

GAPDH replicate 1

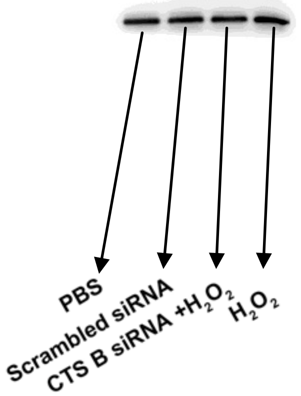

CTSB replicate 2

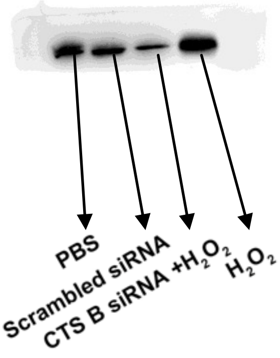

GAPDH replicate 2

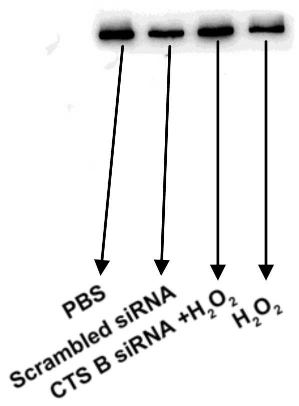

Figure 9 D

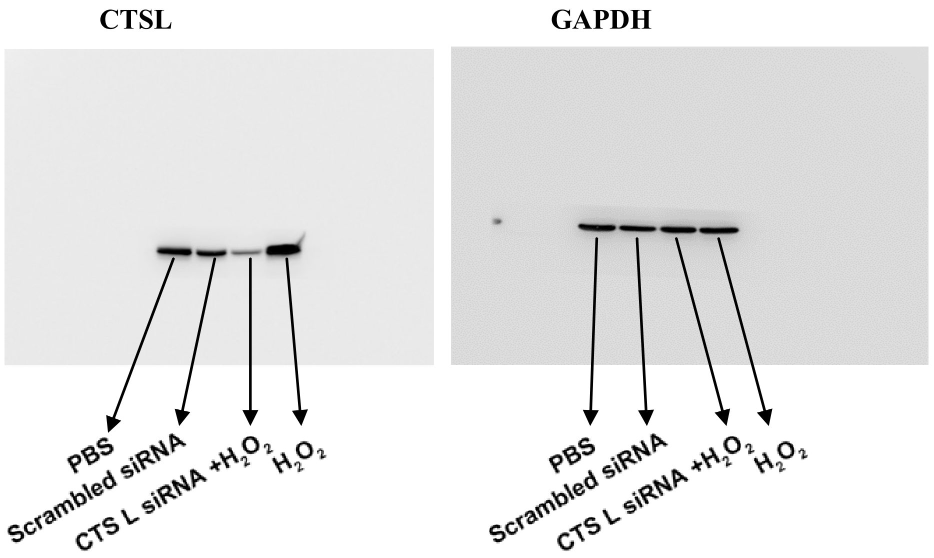

CTSL replicate 1

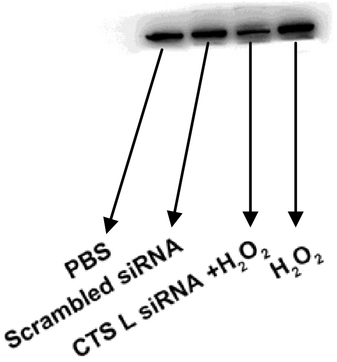

GAPDH replicate 1

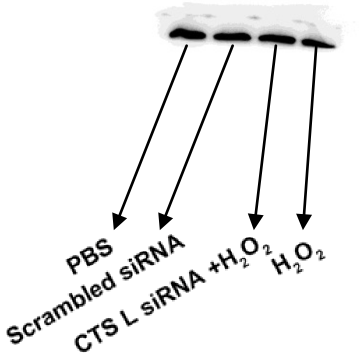

CTSL replicate 2

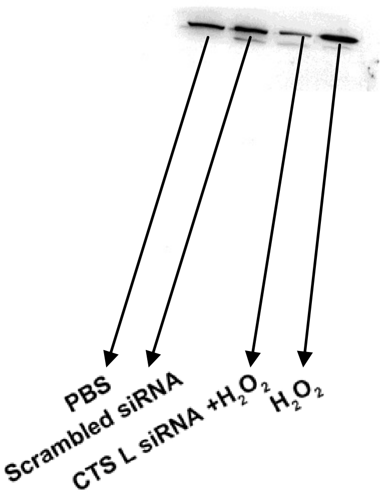

GAPDH replicate 2

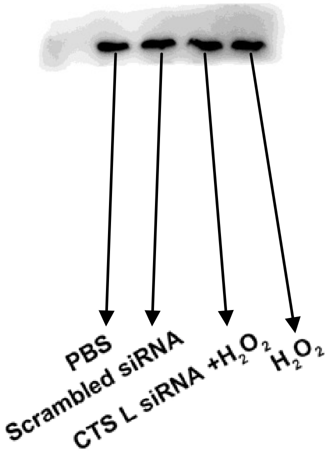

Supplement: Supplementary file 1 — Supplementary material 1 (PDF 542 KB) [file 12985_2023_1964_MOESM1_ESM.pdf]
